# Effects of insemination and blood-feeding on locomotor activity of wild-derived females of the malaria mosquito *Anopheles coluzzii*

**DOI:** 10.1186/s13071-021-04967-0

**Published:** 2021-09-07

**Authors:** Amadou S. Traoré, Angélique Porciani, Nicolas Moiroux, Roch K. Dabiré, Frédéric Simard, Carlo Costantini, Karine Mouline

**Affiliations:** 1 MIVEGEC, Univ Montpellier, CNRS, IRD, Montpellier, France; 2 Institut de Recherche en Sciences de La Santé (IRSS), Bobo-Dioulasso, Burkina Faso; 3 Agro Paris Tech, Institut Des Sciences Et Industries du Vivant Et de L’environnement, Université Paris-Saclay, Paris, France

**Keywords:** *Anopheles coluzzii*, Field, Locomotor activity, Daily rhythms, Insemination, Blood and glucose intakes, Diversity, Chronotypes, Burkina Faso, Residual malaria transmission

## Abstract

**Background:**

Behavioural shifts in the canonical location and timing of biting have been reported in natural populations of anopheline malaria vectors following the implementation of insecticide-based indoor vector control interventions. These modifications increase the likelihood of human-vector contact and allow mosquitoes to avoid insecticides, both conditions being favourable to residual transmission of the malarial parasites. The biting behaviour of mosquitoes follows rhythms that are under the control of biological clocks and environmental conditions, modulated by physiological states. In this work we explore modifications of spontaneous locomotor activity expressed by mosquitoes in different physiological states to highlight phenotypic variability associated to circadian control that may contribute to explain residual transmission in the field.

**Methods:**

The F_10_ generation progeny of field-collected *Anopheles coluzzii* from southwestern Burkina Faso was tested using an automated recording apparatus (Locomotor Activity Monitor, TriKinetics Inc.) under LD 12:12 or DD light regimens in laboratory-controlled conditions. Activity recordings of each test were carried out for a week with 6-day-old females belonging to four experimental treatments, representing factorial combinations of two physiological variables: insemination status (virgin vs inseminated) and gonotrophic status (glucose fed vs blood fed). Chronobiological features of rhythmicity in locomotor activity were explored using periodograms, diversity indices, and generalized linear mixed modelling.

**Results:**

The average strength of activity, onset of activity, and acrophase were modulated by both nutritional and insemination status as well as by the light regimen. Inseminated females showed a significant excess of arrhythmic activity under DD. When rhythmicity was observed in DD, females displayed sustained activity also during the subjective day.

**Conclusions:**

Insemination and gonotrophic status influence the underlying light and circadian control of chronobiological features of locomotor activity. Overrepresentation of arrhythmic chronotypes as well as the sustained activity of inseminated females during the subjective day under DD conditions suggests potential activity of natural populations of *A. coluzzii* during daytime under dim conditions, with implications for residual transmission of malarial parasites.

**Graphical abstract:**

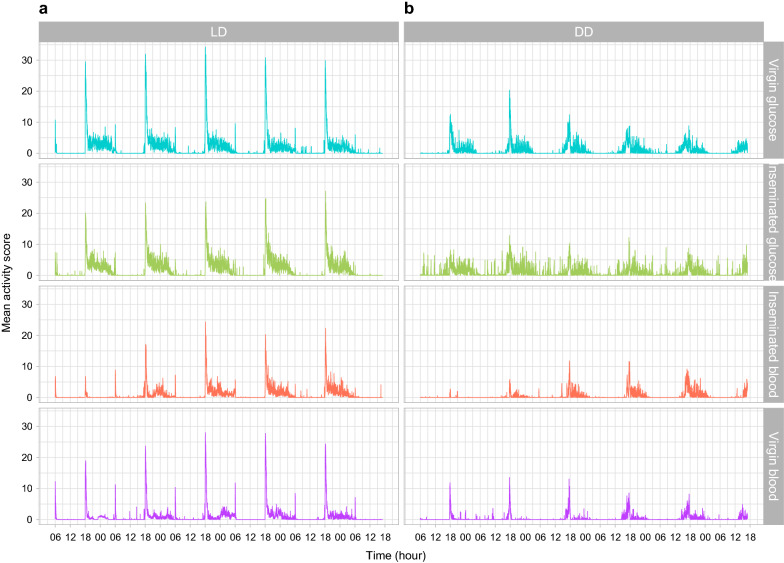

**Supplementary Information:**

The online version contains supplementary material available at 10.1186/s13071-021-04967-0.

## Background

The temporal organisation of behaviour is among the most important functional characteristics of organisms in the living kingdom. The rhythmical occurrence of individual behaviours at specific times of the day or of the year is functional to survival and reproduction, increasing fitness at the individual and population levels [1–4].

Mosquitoes are hematophagous insects, including among the most important vectors of human pathogens such as malarial parasites, *filariae* and several arboviruses such as yellow fever, dengue, Zika, or chikungunya. In haematophagous insects, many behaviours have been demonstrated to show a marked circadian feature, such as locomotor activity [5–8], mating [9, 10], oviposition [9, 11, 12], ecdysis [13], metamorphosis [14], sugar feeding [15, 16], and blood feeding [15, 17]. It is during blood feeding that mosquitoes pick up or transmit viruses or parasites, thus making this behaviour a key component of the pathogen’s transmission cycle [18].

Female mosquitoes go through different physiological states after emergence and during their adult life. Hence, after emergence, virgin females will seek a source of carbohydrates to accumulate energy before looking for a mate [19]. Energy enables them to engage in reproductive behaviours later on [20, 21]. Only after being inseminated, which generally happens once in their lifetime, will females start seeking a blood meal [22]. A period of a low level of activity (i.e. “resting”), which corresponds to a phase of digestion of the blood meal and maturation of the ovaries, follows blood feeding. Only after complete maturation of the oocytes the female is ready to engage in her quest for a suitable water collection where to lay eggs. After oviposition, another gonotrophic cycle (i.e. the cyclical repetition of the sequence of the physiologically controlled blood-feeding, resting, and oviposition behaviours) is engaged, and this is repeated throughout the female’s lifetime. Embedded within the infradian rhythmicity of the gonotrophic cycle, which follows a sequence of gated physiological changes, each of these behaviours is usually associated with circadian periodicities modulated by diel changes in peripheral receptors’ sensitivity to key stimuli [6, 23, 24] or changes in behavioural responsiveness [25–27], but above all the overt expression of behaviours is regulated by the circadian rhythmicity of general activity under the control of molecular clocks [28, 29].

For any given species or sex [10], spontaneous locomotor activity has been shown to depend on endogenous physiological conditions, most notably upon insemination status, nutritional status [9, 16, 28–32], or the nature of the meal [9, 16, 30, 31] as well as on genetic background such as the presence of insecticide resistance alleles [33]. Exogenous biotic or abiotic factors such as light and temperature [34, 35] or parasites [36, 37] can contribute to the modulation of spontaneous activity. Both endogenous and environmental factors operate through their effects on the expression of molecular clock genes. For example, it has been shown in *Lutzomyia longipalpis* and *Drosophila melanogaster* that a reduction in locomotor activity after a blood meal is correlated with decreased levels of expression of the circadian gene *Timeless* (TIM) [8]; likewise, a correlation between reduced locomotor activity after a blood meal and decreased levels of circadian *period* (PER) gene expression has been demonstrated in other hematophagous insects by Gentile et al. [38].

Recently, changes in the temporal profile of blood-feeding and associated behaviours have been reported in natural populations of some anopheline species characterised by anthropophilic feeding preferences. Most of the species concerned are malaria vectors in tropical Africa, i.e. members of the *Anopheles gambiae* and *Anopheles funestus* species complex and group, respectively, that are characterised by peak biting activity late at night, usually between midnight and dawn. There are also a few reports concerning species with more crepuscular feeding habits [39]. Evidence suggests that these temporal changes have occurred after the large-scale deployment of insecticide-treated bed nets to control malaria transmission, presumably in response to selective pressures shifting the blood-feeding activity of these species towards temporal windows when the preferred host is more accessible [39–43]. It is still unclear whether such temporal restructuring of blood-feeding may represent an evolutionary adaptive response and/or a form of behavioural plasticity. Regardless, there is an urgent need to understand the nature of these temporal shifts in behaviour, as the efficacy of vector-borne disease control programmes relies on the occurrence of blood-feeding at night-time when humans are protected by treated bed nets.

To understand and predict evolutionary responses of complex behaviours, it is necessary to dissect the behavioural sequences underlying such complexity to identify those traits that are amenable to evolutionary analysis. For example, to apply quantitative genetic approaches to behavioural evolution, one needs to identify and ‘extract’ the individual phenotypic values of those traits that underlie the complex behaviour under investigation; ultimately, one wants to quantify variation within and between individuals for those traits. The measurement of individual differences, the determination of the causes of the differences, and the assessment of the fitness consequences of such differences are the essence of quantitative genetic analyses of behavioural evolution [44].

Here we use general locomotor activity in the malaria vector *Anopheles coluzzii* (a member of the *A. gambiae* species complex) as a model of a complex behaviour with the aim to identify the underlying quantitative traits describing the temporal organization of the overall locomotion phenotype observed with an automatic recording apparatus. We investigated the effects of male insemination and blood-feeding on spontaneous activity. These factors are known to affect mosquito locomotor activity [9, 30, 36, 45], but differences have not been characterised quantitatively for the underlying traits and in early generations of wild-derived malaria vector populations.

## Methods

### Mosquito colony

Two hundred female anopheline mosquitoes were collected using a mouth aspirator in the village of Bama (11°22′ N, 4°25′ W), southwestern Burkina Faso. Individuals that were morphologically identified as *A. gambiae* sensu lato using the identification keys of Gillies and De Meillon [46] were separated into gonotrophic classes according to abdominal appearance. Mosquitoes that were fully engorged with blood, half-gravid, or gravid were transferred into large cages and then transported to the IRSS insectary facility in Bobo-Dioulasso, about 30 km away from Bama. After a period spent in standard insectary conditions (27  ±  2 °C, 70  ±  5% RH, LD 12:12 cycle) to ensure that most individuals were ready to lay eggs, mosquitoes were isolated in plastic cups filled with 30 ml tap water to allow oviposition. Those individuals that laid eggs were then killed for further molecular characterization of species status following the protocol of Santolamazza et al. [47].

From the results of the molecular identifications, only eggs from *A. coluzzii* females bearing the susceptible allele of kdr (L1014L) were retained; eggs were transferred into trays (30 cm × 20 cm) containing 1 l tap water and then reared following standard colony maintenance protocols, as previously described by Ruiz et al. [48]. The offspring of 108 *A. coluzzii* constituted the F_0_ founders of our working colony, which was established in May 2018; the experiments took place from September to November 2018. We worked on the tenth generation of mosquitoes after their introduction in our insectary. This was the time that had elapsed since the collection from the field and the establishment of the working colony, which was necessary to select for insecticide-susceptible *A. coluzzii* only, to allow females to adapt to our insectary routines, to obtain successful and optimal blood-feeding during the day, to optimise our feeding protocol during the recording to increase females survival in the tubes, and to reach the number of offspring needed to create our working groups simultaneously.

### Experimental design and conditions

The objective of the experiment was to compare the activity of virgin or inseminated females after they had had a meal of either blood or glucose. Four treatment arms were constituted: (i) virgin females fed with 5% glucose; (ii) inseminated females fed with 5% glucose; (iii) inseminated females fed with blood; (iv) virgin females fed with blood. The first three treatments reproduce the usual sequence of physiological changes occurring after pupal metamorphosis and adult emergence in natural populations, while the latter arm is quite rare in nature [14]. However, it was included to look at the effects of insemination and nutritional status in accordance with a 2  ×  2 factorial experimental design.

**Fig. 1:**
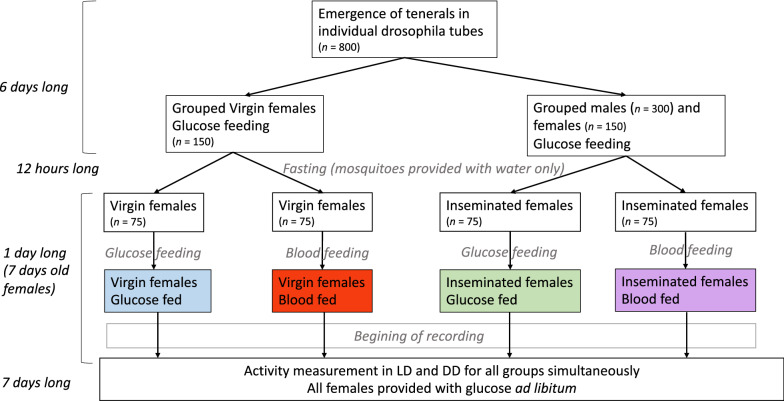
Schematic and summarised representation of our working groups creation with associated timelines and sample sizes

Experimental treatments were constituted as follows (Fig. 1). First, it was necessary to ensure that males and females emerged separately to produce the treatment arms for virgin females. Thus, 800 pupae from the colony were individually placed in plastic tubes (*L*  =  9.3 cm, *D*  =  2.5 cm) plugged with a cotton wool plug and containing 1 ml deionized water. The tubes were placed in a wooden box (50 × 50 × 50 cm) to entrain the test mosquitoes to a 12 h/12 h LD cycle with 11 h full light, 11 h darkness (0 lx) and 1 h linear dawn [from 05:00 (ZT11) to 06:00 h (ZT12)] and 1 h linear dusk [17:00 h (ZT23) to 18:00 h (ZT0)] transitions. In LD regimen, the time of day is reported in 24 h Zeitgeber time (ZT) with 18:00 h (ZT24/0) defined as time of lights off and 06:00 h (ZT12) defined as time of lights on. Light conditions were obtained using a customised system composed of one LED (Vision-EL^®^ GU5.3, 12 V, 4000 °K, 530 lumens, 6-55 W) controlled by a TC420 Programmable LED Time Controller Dimmer (20A, 12 V–24 V, Word uniqueen International Trade Co., Ltd., China).

As soon as tenerals (i.e. newly emerged, immature adults) emerged, two groups were established: virgin and inseminated females. To obtain the latter, 300 males and 150 females were mixed for 6 days in a 30  ×  20  ×  20-cm cage to allow females to mate. At the same time, to constitute the virgin treatment arms, another group of 150 females were placed in a separate cage of the same size and left for 6 days to ensure that mosquitoes from both groups had the same age in the experimental tests. During this time, mosquitoes from both cages were allowed to feed ad libitum on cotton soaked with a 5% sterile glucose solution (5% Glucose Solution for Injection USP, Shandoung Qidu Pharmaceutical Co., Ltd.).

After day 6, males were discarded and all females were starved for 12 h by replacing the glucose-soaked cotton ball by one soaked with water only. Each of the two groups was then subdivided into two subgroups of equal sample size (75 females). One subgroup from each of the two groups was allowed to feed for 30 min on a restrained rabbit, whereas the other was allowed to feed again on a 5% glucose solution. Only fully engorged females were further considered. They were offered a 5% glucose solution right after the blood feeding.

The activity recording began 1 h after blood feeding, and any care or handling of mosquitoes was performed to avoid creating potential, unwanted, zeitgebers that could modify the initially entrained activity. The process of group creation is summarised in Fig. 1.

### Locomotor activity recording

Females from all subgroups were individually introduced into Plexiglass *Drosophila* tubes (*L*  =  9.3 cm, *D*  =  2.5 cm) provided with an ad libitum 5% glucose solution (a cotton plug soaked with 5–7 ml 5% sterile glucose was placed at the bottom of the tube). Tubes were inserted into the holding grid of seven Locomotor Activity Monitors (LAM, TriKinetics, Waltham, MA) to detect and measure spontaneous activity automatically. LAMs are routinely employed in chronobiology [8, 10, 49–52] and have been used in studies of mosquito activity [10]. LAMs automatically record activity on a connected computer when a mosquito breaks an infrared beam across the centre of each holding tube. Each LAM received two control tubes (empty tubes lacking the mosquito within), and in all four treatment arms, treatment tubes and their positions in the LAM grid arrangement were chosen at random using the function “random” in the “Excel” software.

Because some artefacts were detected in control tubes from three out of the seven LAMs (originating from the same batch order), the external surface of all tubes was covered with a white rubber band, except for a 2-cm gap centred on the IR emitter/detector system. This decreased artefacts without impacting activity recording (data not shown). Each LAM was placed inside a hermetic wooden box (50  ×  50  ×  50 cm) in the insectary to prevent contamination from potential light pollution from the surroundings. Environmental conditions and photoperiod were identical to those previously described for rearing, except for two LAMs that were submitted to a constant dark (DD) regimen to measure free-running circadian parameters. Activity was recorded every minute (i.e. at a frequency of 60^–1^ Hz) for 7 consecutive days.

### Insemination status and body size

At the end of the recording period, mosquitoes were killed by placing the holding tubes at − 20 °C for 30 min. To control for the potential confounding effects of body size upon differences in locomotor activity among treatments, we used wing size as a proxy of body size [53]. The right wing from each female was dissected under a binocular, and digital photographs of the wings of all tested mosquitoes were taken. The distance from the axillary incision (*alula*) to the apical margin [54] was measured using ImageJ software [55] and this measure used as a proxy of wing size.

Females from the inseminated treatment arms were further processed according to the protocol described by Tripet and collaborators [56] to characterise their insemination status. Individual mosquitoes were stored in microtubes containing 70% (v/v) ethanol for 4 days; thereafter, their 6th abdominal segment was dissected under a binocular and separated from the rest of the carcass; the spermatheca was isolated and its envelope delicately torn apart to release the sperm bundle, which was observed under 35  ×  magnification. If sperm was found inside the spermatheca, the female was scored as being inseminated. Females from the inseminated treatment arms that failed to yield sperm were scored as non-inseminated and discarded from further analyses.

### Data analysis

The LAM output file returns individual counts of beam breaks per unit time. We call this variable the activity score of each mosquito at a given time. It is assumed that activity scores are proportional to locomotor activity, which is unobserved with this experimental setup.

Activity records from the first and last day of each experimental run were not considered as they were deemed, respectively, a period of acclimation when mosquitoes may not display their normal behaviour [10] or a period of significant decrease in overall activity due probably to a combination of factors such as energy shortage or ageing. Individuals whose actogram showed 24 h of continuous inactivity were considered dead and discarded from analysis.

We examined the propensity of individuals at showing rhythmicity in spontaneous activity by calculating their chi-square periodograms [57]. Statistically significant peaks in the periodogram informed about which periods are associated with significant rhythmicity, above average stochastic noise. From chi-square periodograms, we extracted the significant frequencies (periods) from activity signals under LD regimen and identified the free-running circadian period lengths in DD regimen. For *Anopheles* mosquitoes, the fundamental free running period is generally around *τ*  =  23  ±  1 h on average, showing variability dependent on species, strain, or sex [10]. Therefore, we considered only values in the range of *τ*  =  23  ±  2 h. Few atypical individuals displaying circadian periods departing > 2 h from 23 h were discarded. Fundamental circadian period lengths were analysed according to food source (glucose vs blood), insemination status (inseminated vs virgin), photoperiod regimen (LD vs DD), and corresponding interactions, using multiple linear regression modelling.

In addition to the fundamental period (i.e. the period length in the range of *τ*  =  23  ±  2 h), we also considered, for each individual, those periods that could be recovered from our activity score series and assessed as significant from the periodograms. These periods were of lengths *τ*  =  6, 8, 12, 16, 18, 20, and 30 h. For each individual, the presence or absence of each of these periods was recorded as a binomial variable (0  =  absence, 1  =  presence). Hence, each individual was characterised by a specific binary code that is represented at a given frequency that may differ between groups. We evaluated the periods’ diversity (which encompasses the qualitative as well as the quantitative aspects described above) for each group and both light regimens by computing the Shannon diversity index [58]. For a given group, lower diversity index indicates the predominance of a few combinations of periodic components in mosquito’s activity among individuals of each group. Conversely, higher diversity indicates that, in the considered group, individuals differed (more) among them in their combination of significant periodicities.

The mean activity score (i.e. the number of times that the mosquito crossed the infra-red beam at the recording frequency of 60^–1^ Hz, averaged across individuals in each treatment arm) was used to display actograms in LD and DD. This endpoint provided the general shape of the temporal structure of locomotor activity during the whole test period, allowing visual comparison among treatments.

Activity per night and per hour of the night in each treatment arm provided the mean nightly and mean hourly activity endpoints, which were used to apprehend the dynamics of locomotion during the course of the experiment. For this task, we modelled individual activity scores recorded between 16:00 h (ZT10 or CT10 in DD regimen) and 07:00 h (ZT1 or CT0 in DD regimen) and expressed at the recording frequency of 300^–1^ Hz (i.e. per 5 min) using negative binomial GLMMs (generalised linear mixed models). For mean nightly activity analysis, we analysed LD and DD activity score data separately according to the insemination status (virgin vs inseminated), food source (glucose vs blood), recording day (coded as a factor value), and their interactions. For hourly activity analysis, due to convergence issues, we split the dataset to analyse the effect of insemination in glucose-fed mosquitoes and then the effect of blood-feeding in inseminated females in both the LD and DD regimens on day 3. Therefore, the explanatory variables of the GLMMs were the insemination status or the feeding source, the hour of the night (coded as a factor value), and corresponding interactions. To deal with possible auto-correlation in the data due to repeated measurements, intercepts and slopes between days (or hours) were allowed to vary by individuals.

To analyse activity during and just after the transition from photo- to scotophase, we identified and compared the onset of activity and the time of peak locomotory activity. The onset of activity was expressed as the time (in hours and minutes) when sustained locomotory activity starts during the artificial dusk. Start of sustained activity was arbitrarily defined as the time when each mosquito produced at least  ≥  1 beam breaks per minute for 3 consecutive min. The time of peak locomotory activity was expressed as the time (in hours and minutes) when each mosquito activity score reached the maximum value during the artificial dusk. Onset and time of peak activity were analysed separately between LD and DD using mixed-effect linear models with food source, insemination status, days of recording, and interaction between food source and insemination status as explanatory variables and individuals as random intercepts.

Actograms were made using the “dplyr” package [59]. Chi-square periodograms were calculated using the “xsp” package [60].

Mixed-effect models were fitted using the “glmmTMB” package [61] in R software [62]. Adequacy of model residuals was checked using the “DHARMa” package [63]. Estimated marginal means were estimated and compared using the “emmeans” package [64]. *Post-hoc* Tukey’s procedures with correction for multiple comparisons were run, when appropriate, to compare the levels of significant factors.

## Results

### Arrhythmic mosquitoes

The total number of rhythmic or arrhythmic individuals broken down by treatment and photoperiod is given in Table 1. Of the 200 individuals tested (140  +  60 in LD and DD, respectively), 8 (4%) were discarded because they did not survive across the 7 days of testing, and 27 (13.5%) were females that were not inseminated despite sharing the same cage as the males. Arrhythmic individuals accounted for  ~ 3% of the sample in LD and  ~ 16% in DD, the difference being statistically significant (*G* test of independence with Williams’ correction *G*  =  5.57, *df*  =  1, *p*  =  0.02). Arrhythmic individuals were especially overrepresented in the group of inseminated females under DD, regardless of meal type (*G* test of independence with Williams’ correction *G*  =  11.12, *df*  =  3, *P* =  0.01).

**Table 1:** Total number *n* of individuals included in the analysis according to treatment and photoperiod

Insemination status	Food source	Light regimen	*n* of rythmics	*n* of arrhythmics	*n *total
Virgin	Glucose	LD	32	1	33
Virgin	Glucose	DD	15	0	15
Inseminated	Glucose	LD	24	1	25
Inseminated	Glucose	DD	11	4	15
Inseminated	Blood	LD	21	1	22
Inseminated	Blood	DD	11	3	14
Virgin	Blood	LD	35	1	36
Virgin	Blood	DD	14	1	15
Total		LD	113	4	116
Total		DD	50	8	58

The “Arrhythmic” column shows the number of individuals in each group that display arrhythmic activity according to chi-square periodograms and were further discarded from activity representations or analysis

### Impact of body size

Wing size did not differ among treatments (linear model; cf. Additional file 1) and was not correlated with locomotor activity (Pearson correlation; cf. Additional file 1). Thus, in what follows, wing size was not considered a potential confounding variable and was excluded from further analyses.

### Description of overall activity phenotypes

**Fig. 2:**
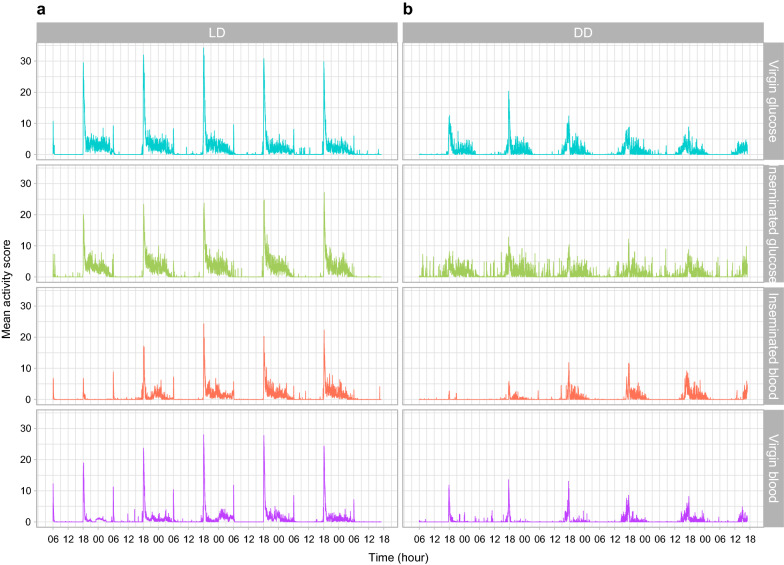
Mean activity score (beam breaks min^−1^) during 5 days of recording for *Anopheles coluzzii* females for each of the four physiological conditions tested under two light regimens: **a** light:dark 12:12 (LD); **b** dark:dark (DD)

As anticipated, visual inspection of the actograms suggested distinct daily mean activity patterns among treatments (Fig. 2).

#### Activity under LD vs DD

The intensity of diel activity was on average lower in DD than LD. Daily patterns were bimodal in LD and mostly unimodal in DD. All treatments showed the highest peak of activity following the lights-off transition at 18:00 h (ZT12) in LD regimen and at or before 18:00 h (CT12) in DD, depending on the subjective day considered. The amplitude of this peak was clearly greater under LD than DD conditions. This lasted for 1–2 h, after which activity substantially decreased and remained at an intermediary level until the lights-on transition in LD regimen. At lights on at 06:00 h (ZT24/0) in LD, there was a peak of activity that disappeared under DD regimen. Between these two temporal points, i.e. during the day or the subjective day, there were  few minor activity peaks of low intensity, except under DD conditions where inseminated glucose-fed females seem more active than any other group (Fig. 2, and see below).

**Table 2:** Shannon diversity index *H* for the frequency of significant periods observed in individual’s chi-square periodograms for each treatment group under LD and DD regimen, as shown in Additional file 1: Table S1

Insemination status	Meal	H	Light regimen
Virgin	Glucose	1.75	LD
Inseminated	Glucose	1.21	LD
Inseminated	Blood	1.06	LD
Virgin	Blood	2.13	LD
Virgin	Glucose	1.49	DD
Inseminated	Glucose	1.12	DD
Virgin	Blood	1.50	DD
Inseminated	Blood	1.77	DD

#### Activity of virgin vs inseminated females

Under LD conditions, virgin females appear to display a greater initial peak of activity, after which the activity is greater for inseminated females than for virgins (Fig. 2, panel a). Moreover, the presence of the peak at lights on (06:00 h (ZT24/0) is less intense or completely disappears in inseminated females. The largest difference between both physiological statuses is that under DD conditions (Fig. 2b), inseminated glucose-fed females display consistent and sustained activity during the subjective day, where their virgin counterparts seem still. This trend is not observed for the blood-fed group.

#### Diversity index

The Shannon diversity index (*H*) is a quantitative measure of the diversity of a specific factor, in this case, the overall locomotor activity. Here, and from our analysis of the periodograms, we consider that the locomotor activity factor is actually expressed by the mosquito as a temporal sequence of different frequencies. Among these frequencies, there is the fundamental and well described one of around 24 h, but not only; other secondary frequencies are significantly expressed as well, above average stochastic noise. As previously described, the *H* index is computed from the presence/absence binary table of each of the eight frequencies, for each individual, and for each group of physiological status (Additional file 1). For example, in this table, arrhythmic individuals have the null value for all the identified periods.

Under LD conditions, we evidenced a higher diversity of frequencies (i.e. periods) among virgin blood-fed females compared to inseminated ones (Table 2). When we considered inseminated females only, we observed that the virgin females had a twice greater diversity index than the inseminated ones (*H*virgin  =  2.08, *H*inseminated  =  1.24). Between blood- and glucose-fed females, there is a trend for more diversity for the blood-fed group (*H*blood  =  2.00, *H*glucose  =  1.72). Under DD regimen, the same trend can be observed when comparing inseminated and virgin females, with a higher diversity index for the latter group (*H*inseminated  =  1.19; *H*virgin  =  1.81). For all groups except the “Inseminated Blood” one, *H* values are higher in LD than DD (Table 2).

### Mean nightly and hourly activity

**Fig. 3:**
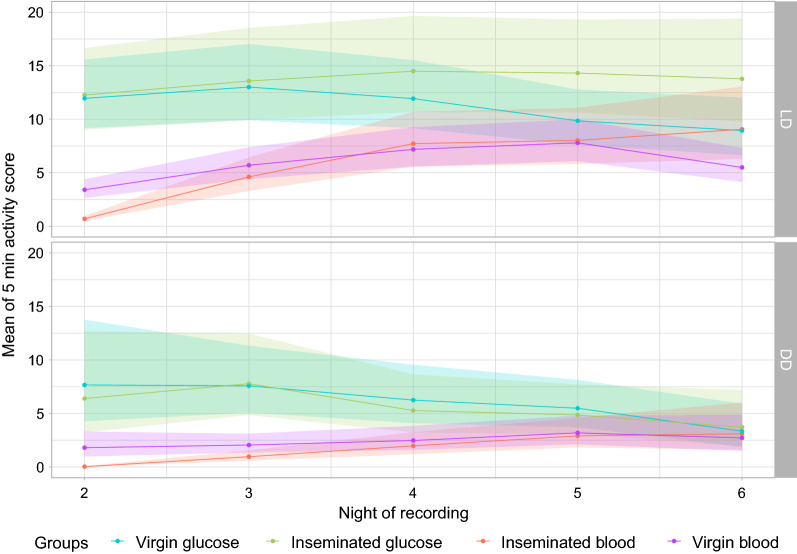
Mean nightly activity predicted by the models under light:dark (LD, upper panel) and dark:dark light regimens (DD, lower panel). Points correspond to model estimated marginal means and color shades represent 95% confidence intervals

To allow models to converge for statistical comparisons between the groups’ locomotor activities, we considered activities recorded only during the night in LD and subjective night in DD, expressed per hour, from 16:00 h (ZT10) to 06:00 h (ZT0/24). Mean nightly activity scores as predicted by the GLMMs are represented in Fig. 3.

The 2nd day of recording and under LD and DD conditions, mean nightly activity of blood-fed females was significantly lower than that of glucose-fed ones, regardless of their insemination status (detailed multiple comparison statistics available in Additional file 1, “nightly activity”). If we consider the group of blood-fed females only for this same day, virgins displayed a higher activity level than their inseminated counterparts [comparison between blood-fed inseminated and virgin females; for LD conditions, rate ratio (RR)  =  0.20, 95% CI  =  (0.11–0.35), *P* <  0.001; for DD, RR  =  0.018, 95% CI  =  (0.004–0.068), *P* <  0.001] but this difference disappears for the consecutive ones (see Additional file 1). Significant differences remained between glucose- and blood-fed groups until the 4th day of recording under LD conditions. The same trend was observed for DD females, but the differences were only significant for the 3rd day (see Additional file 1).

For each group, we performed a comparison of activity between consecutive nights and found that under the LD regimen activity remained constant only in the glucose inseminated group (see Additional file 1), while it significantly decreased between the 5th and 6th nights of recording for the virgins [comparison between night 5 and 6 for the virgin glucose group, RR  =  2.06, 95% CI  =  (1.08—3.91), *P* =  0.02]. For the blood-fed group, only inseminated females displayed significantly different activity between consecutive days, these days being the 2nd, 3rd, and 4th days of recording [comparisons of the activity levels between nights 2 and 3, RR  =  0.033, 95% CI  =  (0.013–0.082), *p*  <  0.001; between nights 3 and 4, RR  =  0.44, 95% CI  = (0.25—0.78), *p* < 0.001]. Comparison between nights under DD conditions revealed significant differences for blood-fed inseminated females for the same nights than under LD [comparisons of the activity levels between nights 2 and 3, RR  =  0.034, 95% CI  = (0.013–0.085), *P*  <  0.001; between nights 3 and 4, RR  =  0.48, 95% CI  =  (0.28–0.84), *P* = 0.0036]. However, although the same trend appears, no significant differences could be seen between nights for DD glucose-fed inseminated females (Additional file 1).

**Fig. 4:**
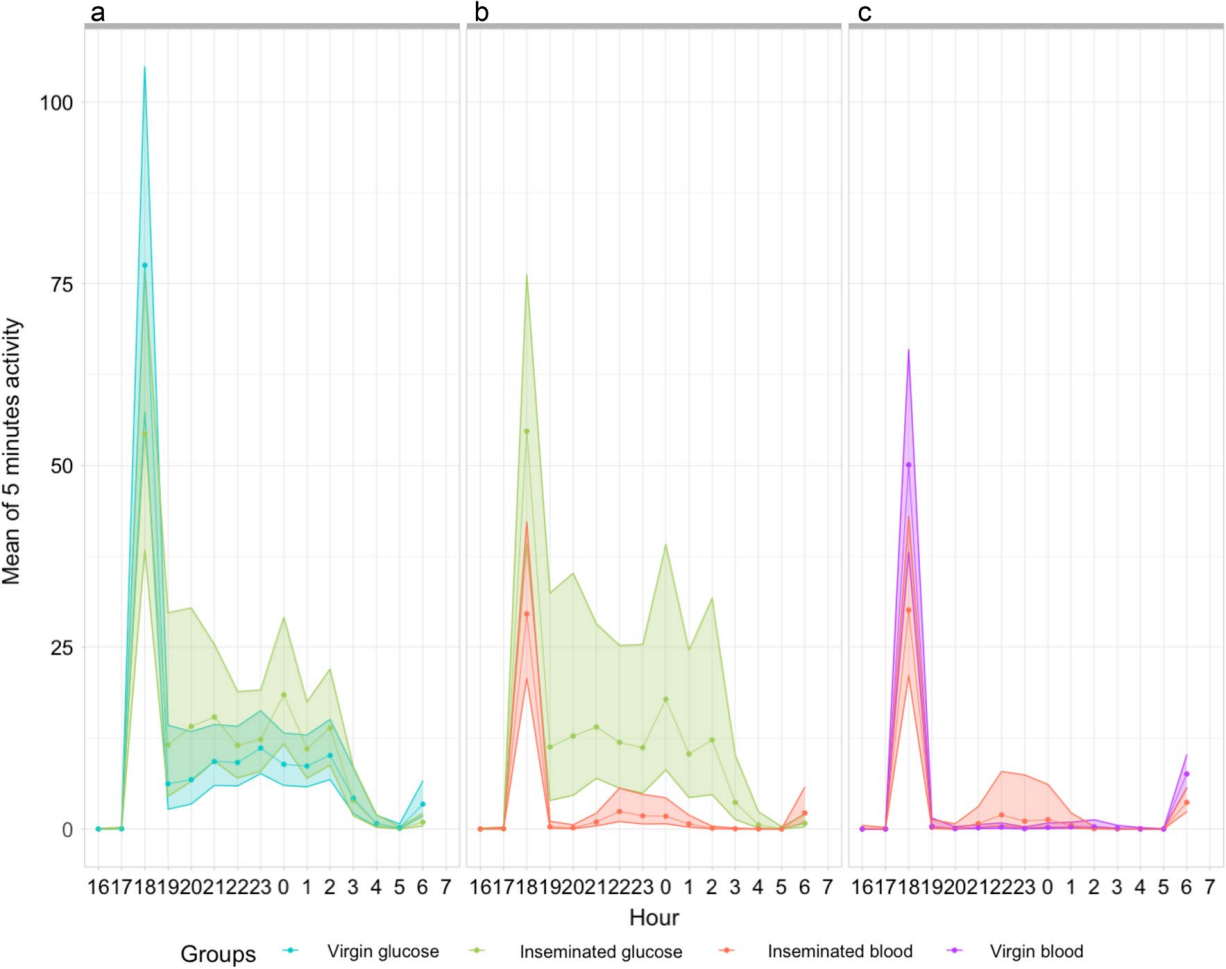
Mean hourly activity during the 3rd night for glucose-fed females (**a**), inseminated females (**b**) and blood-fed females (**c**) in LD regimen. Because the effects of insemination and food source were analyzed separately, confidence intervals for activity of inseminated glucose-fed females may vary between panels **a** and **b**. Points corresponding to the model estimated marginal means and color shades represent 95% confidence interval

As the differences between the groups were significant on the 3rd night of recording (Fig. 3) under both LD and DD conditions, we wanted to investigate this trend more deeply by analysing the mean hourly activity predicted by the model for this specific night (Fig. 4). Three paired comparisons were performed to determine the impact of the food source or the insemination status on the hourly activities. For the glucose-fed group, which allowed a comparison between insemination statuses (Fig. 4a), virgin females showed a tendency, although not significant on this night of recording, toward higher activity than inseminated ones following the day:night transition [18h00/ZT12, rate ratio (RR)  =  0.70, 95% CI  =  (0.44; 1.11), *P* =  0.13], but also at night/day transition, and this trend was here significant [06h00/ZT0, RR  =  0.27, 95% CI  =  (0.09; 0.76), *P*  =  0.014] (Fig. 4a; Additional file 1). For the blood-fed group (Fig. 4c), activity is significantly higher for virgin females than for inseminated ones at both transitions [18h00/ZT12, rate ratio (RR)  =  0.60, 95% CI  =  (0.38; 0.94), *P* =  0.02, 06h00/ZT0, rate ratio (RR)  =  0.48, 95% CI  =  (0.25; 0.81), *P*  <  0.01]. Between these light transitions, activity of inseminated females has a tendency to be higher (Fig. 4), the difference being significant at 00:00 h only for glucose-fed females [(ZT18), RR  =  2.07, 95% CI  =  [1.13; 3.8], *P* =  0.018] and at 22h00/ZT16 [RR  =  7.42, 95% CI  =  (1.24; 44.29), *P* =  0.027] and 23h00/ZT17 [RR  =  22.87, 95% CI  =  (1.80; 290.21), *P* =  0.015] for blood-fed females. Differences are marginally significant at 00h00/TZ12 [RR  =  5.86, 95% CI  =  (0.82; 41.98), *P* =  0.07] and 03h00/ZT21 [RR  =  0.08, 95% CI  =  (0.006; 1.09), *P* =  0.058]. When comparing the same groups under the DD regimen, there was no evidence for differences in mean hourly activity (Additional file 1: Table S1).

Activity of virgin glucose-fed females started to decrease between the 4th and 5th nights of recording while that of inseminated ones remained constant (see above). We further analyzed hourly activities between the same groups for the 5th night and found significant differences for the major part of the night (see Additional file 1: Table S1 for all the statistical reports). Inseminated females had significantly higher activity levels than virgin ones at 19h00, 20h00, 22h00, 0h00, 1h00, and 2h00 (i.e. ZT 13, 14, 16, 18, 19, and 20) and marginally significantly higher activity levels at 21h00/ZT15 and 23h00/ZT17). No difference between groups was seen for the dusk transition, but at dawn (06h00/ZT0), virgin females had higher activity levels than inseminated ones.

As expected, the food source induced the greatest differences in hourly activities between inseminated females, with glucose-fed females displaying the highest activity level starting from 18:00 h (ZT12) to 05:00 h (ZT23) (see Fig. 4b; Additional file 1 for hourly comparisons between blood- and glucose-fed inseminated females).

Our models did not allow us to infer the statistical significance of the visual differences observed in Fig. 2 between blood-fed virgin and inseminated females as well as between blood- and glucose-fed inseminated females under DD conditions (Additional file 1: Table S1).

### Period length

**Fig. 5:**
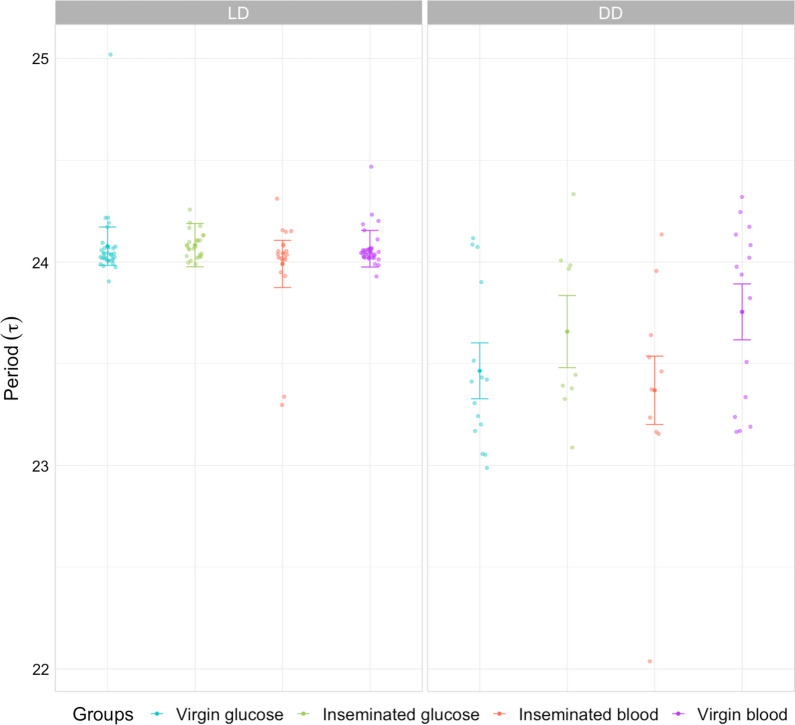
Mean period lengths (±  95% CI) under LD (left) and free-running DD (right) regimen. Dots indicate individual circadian periods as identified using chi-square periodograms (see Materials and methods section)

We compared the period length according to food source and insemination status under both LD and DD regimens. Under LD regimen (Fig. 5), the average period length was ≥ 24 h and not different among groups (see Additional file 1). Under DD regimen (Fig. 5), as expected, all groups showed circadian period length < 24 h. In addition, virgin blood-fed females showed longer period length than both virgin glucose-fed [mean difference (MD)  =  0.29 h (0.03; 0.54), *P* = 0.02] and inseminated blood-fed females [MD  =  0.39 h (0.1; 0.67), *P* =  0.003] but no significant difference was found with inseminated glucose-fed females [MD  =  0.017 h (− 0.16; 0.2), *p * =  0.99]. Although we noticed trends, we were not able to show significant differences when performing meaningful comparisons for characterizing, for instance, specific effects of insemination (comparison between virgin and inseminated physiological groups).

### Times of onset and peak time of twilight activity at sunset and subjective sunset

**Fig. 6:**
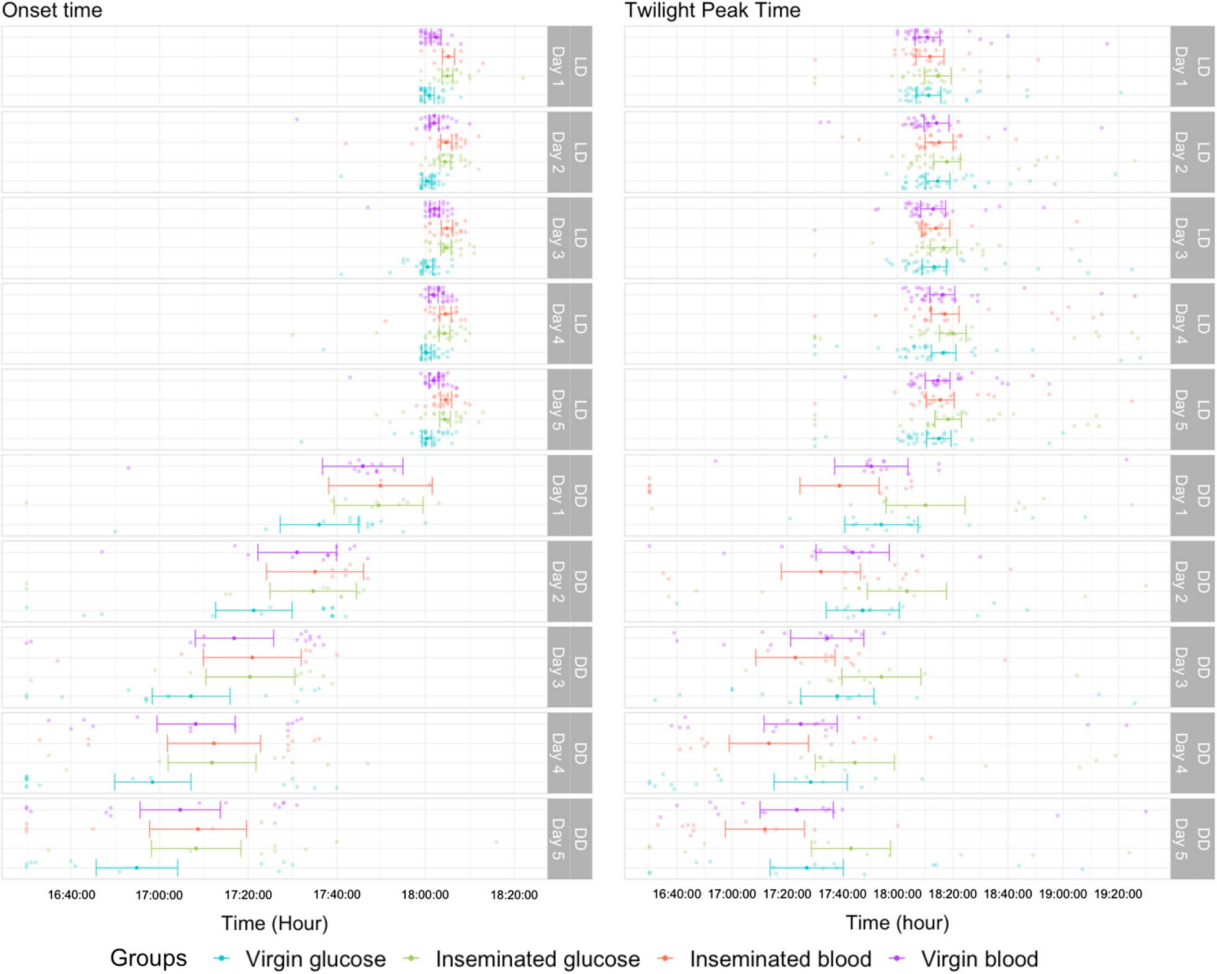
Onset and peak times of twilight activity under different artificial regimens (*LD* light:dark; *DD* dark:dark). The y-axis represents the 5 days of recording and the x-axis represents the time in hours, minutes, and seconds. The soft dots represent raw data and the solid dots represent estimated marginal means computed by the model. Bars represent 95% confidence intervals

When comparing between glucose-fed females under LD conditions, we found that insemination significantly delayed the onset time of activity by 4.03 min on average [95% CI  =  (2.67; 5.39) (*P* <  0.001; Fig. 6)]. The same was observed in blood-fed females with a significant delay for inseminated females of 2.80 min [95% CI  =  (1.30; 4.31) (*P* <  0.001; Fig. 6)]. Blood source also impacted the onset of activity for virgin females only, with glucose-fed females starting to be active 1.54 min earlier than blood-fed ones [95% CI  =  (0.33; 2.74), *P* <  0.01].

Under DD regimen, persisting significant differences are observed when comparing virgin and inseminated females fed with glucose, with an activity for the later starting on average 14.54 min later than the virgin ones [95% CI  =  (3.303; 26.05) (*P* =  0.007;s Fig. 6)].

The time of peak activity has been determined in the restrained time window between 16:30 h (CT10.5) and 19:30 h (CT13.5) for DD regimen and 17:30 (ZT11.5) and 19:30 h (ZT13.5) for LD regimen, encompassing the dusk period where swarming takes place in the fields. By comparing the time of peak activity between physiological groups, we were not able to show any differences under LD regimen (Fig. 6; Additional file 1). However, under DD regimen, blood-fed inseminated females exhibit an earlier peak of activity than glucose-fed inseminated [MD  =  31 min, 95% CI  =  (11; 50), *P* =  3.1  ×  10^–4^] or glucose-fed virgin [MD  =  20 min 95% CI  =  (1.4; 37), *P*  =  0.029] females. Overall, inseminated blood-fed females display the earliest peak of activity while inseminated glucose ones peak the latest.

## Discussion

This study aimed at investigating how fundamental physiological changes induced by mating and blood feeding interact in modulating the spontaneous locomotor activity of females of the malaria mosquito *A. coluzzii*. One of the major differences between this study and previous works addressing this question is the use of early generation progenies issued from females collected from natural populations instead of long-established laboratory strains. This protocol was conceived with the intent to capture the natural diversity of spontaneous activity phenotypes to address questions related to the extent of observable variability and how such variability may lead to evolutionary changes in the circadian temporal organization of behaviours relevant to mosquito-borne disease transmission. There is in fact accumulating evidence that populations of *Anopheles* exposed to mass deployment of insecticide-treated bed nets for malaria control are modifying the timing of biting behaviour toward hours of the day when humans are more readily accessible [39–43]. In doing so, mosquito vectors also reduce the likelihood of coming in contact with insecticide-treated surfaces, thereby reducing the impact of control interventions aimed at reducing transmission of pathogens. In this context of residual transmission of mosquito-borne pathogens, it becomes important to assess the nature and extent of these behavioural changes and the role played by physiological and circadian mechanisms in their determination.

More specifically, this study investigated whether and how combinations of two physiological (virgin vs inseminated) and two nutritional (glucose-fed vs blood-fed) states shape features of daily and circadian rhythms of spontaneous activity. If it is assumed that spontaneous activity imposes a gating mechanism to other behaviours, it can be expected that the study of spontaneous activity can reveal features that can elucidate changes in biting activity. Similar questions were addressed > 60 years ago using classical chronobiological approaches [9, 30]. Under this framework the average behaviour of the experimental populations is of more interest than the behavioural variability of individuals. Because evolution builds upon heritable phenotypic variation to produce evolutionary changes, we aimed at capturing such behavioural variability in activity phenotypes at different levels of biological and temporal organisation to relate: (i) the innate variability in mosquito activity as it is expressed in field populations, (ii) the day-to-day variability in the activity profile of an individual, and lastly (iii) the infra-dian variability in rhythmicity of an individual, whereby activity is broken down in its constitutive oscillations. Hence, an individual’s locomotor profile was characterised as a series of main oscillations in activity strength, each with its own frequency, a sort of barcoding of the activity rhythmicity.

### Activity patterns and insemination status

The spontaneous activity of same-age individuals from the tested physiological groups was measured simultaneously under the same LD and DD environmental conditions. The increased number of inseminated females found arrhythmic under DD and the expression of this phenotype, therefore, are unlikely to be a product of chance alone. To the best of our knowledge, arrhythmicity in spontaneous activity has not been reported in *Anopheles* or in other mosquitoes, and most studies reporting arrhythmicity in other insects are from experimental mutants in *Drosophila* that have been knocked down for endogenous clock genes [65–67]. In mosquitoes, disruption of rhythmic behaviours can also be obtained by a constant light regimen (LL), which alters the oscillatory expression of core clock genes resulting in loss of rhythmicity of many behaviours, including biting, flight activity, and oviposition [51]. Interestingly, disruption of clock gene oscillation induced by LL also alters the timing of light attraction/avoidance in *A. coluzzii*, with mosquitoes losing day vs night differences in light-related behaviours [68]. The over-representation of arrhythmic mosquitoes and the subjective day activity of glucose-fed inseminated females under DD mimic these LL-induced phenotypes. The enhanced activity during the subjective day in this group might be due to disruption of mechanisms involved in light-related behaviours. In other words, spontaneous activity of inseminated females during the photophase may be inhibited by light under LD, presumably in a dose-dependent manner. It is not known, however, how and whether light-induced inhibition and arrhythmicity are interrelated [69, 70].

It is not known, in the first instance, whether arrhythmicity in mosquitoes is adaptive or rather maladaptive. In social insects, like ants and honey bees, arrhythmicity appears after mating [71]. The winged females of the ant *Vergomessor pergandei* (Mayr) display arrhythmic behaviour after the mating flight, but only if they actually mated [72]. Loss of rhythmicity is thought to be adaptive in this case, as the future queen ant sacrifices locomotor activity in favour of reproduction under the constant conditions of the underground environment. From an adaptive point of view, it could be argued that after insemination and before blood feeding, *Anopheles* females may be physiologically wired to find a host, regardless of the period of the day. This would ensure that blood feeding occurs as soon as possible, thereby shortening the gonotrophic cycle. The evidence presented in this study that inseminated females express a potential for daytime activity under DD is concordant with this argument. In the field, this daily activity potential may take place, under certain conditions, in dim habitats, e.g. in the rainforest or dark households. Interestingly, this is also concordant with recent reports of diurnal biting activity indoors by afrotropical malaria vectors (Sangbakembi et al. submitted manuscript).

To reproduce and transmit the *Plasmodium* parasites in the process, an *Anopheles* female has to go through a series of sequential events (mate, feed, mature eggs, oviposit) whose outcome depends at least in part on the characteristics of its locomotor activity, from either a quantitative (the strength of activity) or qualitative perspective (the period at which activity takes place). The sequence of events, however, is not completely inflexible, but is somewhat contingent on resource availability and state dependence [73, 74].

By extracting from periodograms the underlying oscillations in activity embedded in the overall locomotor signal, and analysing their diversity by means of the Shannon index, we have shown that the quality of the embedded oscillations changes depending on physiological status. Females that are physiologically at the earlier stages of the reproduction sequence (glucose-fed virgins) show a more diverse oscillatory nature of locomotor activity compared to females that are physiologically in the later stages of the sequence (blood-fed inseminated). In other words, females that have the potential to express a greater number of alternative behaviours, because they are not yet mated and engaged in the gonotrophic cycle, show the more diverse pattern of locomotor rhythmicity compared to females whose physiological status gates locomotion towards completion of the gonotrophic cycle.

From a quantitative perspective, insemination modifies the strength of *A. coluzzii*’s female activity in both LD and DD; this effect is apparent from the decreased levels of the peaks that occur shortly after lights off and lights on under LD, the increase in overall night activity in both LD and DD, a sustained and constant level of activity at night for at least 6 days, and, lastly, from the delayed onset of activity under both light regimens, this delay reaching almost half an hour in some individuals under DD. The modification of activity strength by insemination has been previously observed in *Anopheles* [9, 30] and in other mosquito species [75]. For example, two closely related taxa of the *Culex pipiens* complex, *Cx p. pallens* and *Cx p. molestus*, both display strengthened nightly activity after insemination, and in *Cx p. molestus* daily activity is suppressed so that this taxon becomes purely nocturnal [76, 77]. This behavioural change ensued after mating, but also following the injection of male accessory gland extracts. In *Drosophila*, mating inhibits the daytime siesta of females [78]. Using male mutants lacking a product of the male accessory gland, the sex peptide, these authors demonstrated that this molecule is the “molecular switch” promoting the females’ new activity pattern. Similar approaches could be useful to understand the molecular mechanisms triggering restless phenotypes in *Anopheles* mosquitoes as well and to what extent this phenotype is associated with changes in the circadian control of light-related behaviours.

### Effects of food intake and light regimen

Regarding the effect of blood-feeding, our main results showed that blood meal intake altered the profile and intensity of locomotor activity in *Anopheles*, as has been observed by other authors [9, 31]. Blood meal significantly reduced *Anopheles* activity for up to 72 h, after which an increase in activity was observed. A considerable decrease in activity after blood intake occurs in the wild during resting, which is a phase of digestion of the blood meal and maturation of the ovaries. Moreover, some authors have observed a 72-h inhibition of blood meal [79] or sugar meal [16] search after blood feeding, but not after sugar feeding, in female *Anopheles* [80]. Here again, like for glucose-fed females, we observe a tendency, although not significant, of a more sustained activity along the whole experiment in inseminated females, probably as a consequence of more energy for flight.

Comparison between DD and LD regimens gives hints about the light dependency of certain phenotypes or their pure circadian origin, or both [10]. Here, we showed that insemination and food regimen provoked phenotypic modifications in the period, onset, and intensity of activity that have a circadian basis. Because so many variables are at play in the expression of rhythmic behaviours in the field, it is not useful to infer what consequences these differences in activity may have in natural populations. However, our findings are in favor of the hypothesis that related molecular changes concern clock core genes or other gene families at play in circadian functions. Candidate genes may be found by emulating studies of *Drosophila*, which is by far the most studied insect regarding its circadian features, at the behavioural and molecular levels. Notably, Harbison et al. [81] recently published a thorough work on genome-wide association study of circadian behaviour in *Drosophila melanogaster*. Their work was based on the Drosophila Genetic Reference Panel. This is a bank of 192 full-sibling inbred lines that has been constructed using field-collected mated females. By interrogating 167 of these lines for their circadian period and rhythmicity index, Harbison and colleagues found 12% to be arrhythmic in concordance with previous observations from natural populations [82]. They found, moreover, an extreme period phenotype of *τ*  =  27 h, identified using periodograms. Interestingly, our findings about arrhythmicity and existence of individuals displaying long periods resonate with these results.

Evidence for genetic control of modifications in biting timing in *Anopheles* has been recently given by Govella et al. [83], who showed that early or late biting have a heritable basis. In *Drosophila*, all the genes found to be associated with peculiar phenotypes, although not obligatorily from the core clock genes, altered circadian features of wild-type flies when knocked down. Hence, examination of *Drosophila* orthologue candidate genes in *Anopheles* that have been characterised in distinct chronotypes might be a first step to understand the molecular mechanisms at play and their potential to respond to selective pressures exerted by vector control interventions.

## Conclusions

In this study, we have measured under controlled laboratory conditions the spontaneous locomotor activity of early generations of field-collected *A. coluzzii* and experimentally created different physiological groups with the purpose to capture the natural diversity of daily and circadian activity phenotypes, while limiting at the same time the impact of confounding environmental and physiological factors.

To capture such diversity, we have applied a statistical framework that controls for sources of interdependence in the data due to repeated measures on the same individuals and at the same time allows investigation of individual contributions to population-level effects. This approach generates richer information that can be exploited towards a more refined understanding of phenotypic diversity and the evolution of circadian activity rhythms.

Such understanding is important to characterise the potential and the mechanisms by which malaria vectors adapt to environmental changes due to anthropogenic selection pressures affecting circadian activity phenotypes, such as insecticide-treated bed nets, with manifest consequences on our ability to acknowledge and mitigate the risks of behavioural resistance in the context of residual malaria transmission.

## Supplementary Information

**Additional file 1: **Among-group comparison statistics of nightly activity, hourly activity, time of onset, period, time of peak activity, and wing size.

## Data Availability

The datasets used and the corresponding R codes created for their analysis could be shared upon request.
